# The Resistance Mechanisms and Treatment Strategies for ALK-Rearranged Non-Small Cell Lung Cancer

**DOI:** 10.3389/fonc.2021.713530

**Published:** 2021-10-01

**Authors:** Yue Pan, Chao Deng, Zhenhua Qiu, Chenghui Cao, Fang Wu

**Affiliations:** ^1^ Department of Oncology, Second Xiangya Hospital, Central South University, Changsha, China; ^2^ Central South University, Changsha, China; ^3^ Hunan Cancer Mega-Data Intelligent Application and Engineering Research Centre, Changsha, China; ^4^ Hunan Key Laboratory of Tumor Models and Individualized Medicine, Second Xiangya Hospital, Central South University, Changsha, China; ^5^ Hunan Key Laboratory of Early Diagnosis and Precise Treatment of Lung Cancer, Second Xiangya Hospital, Central South University, Changsha, China

**Keywords:** anaplastic lymphoma kinase (ALK), TKI - tyrosine kinase inhibitor, resistance, NSCLC, therapy

## Abstract

Anaplastic lymphoma kinase (ALK) is a validated molecular target for non-small-cell lung cancer (NSCLC). The use of tyrosine kinase inhibitors (TKIs) has led to significantly improved survival benefits. However, the clinical benefits of targeting ALK using TKIs are limited due to the emergence of drug resistance. The landscape of resistance mechanisms and treatment decisions has become increasingly complex. Therefore, continued research into new drugs and combinatorial therapies is required to improve outcomes in NSCLC. In this review, we explore the resistance mechanisms of ALK TKIs in advanced NSCLC in order to provide a theoretical basis and research ideas for solving the problem of ALK drug resistance.

## 1 Background

Rearrangement of the anaplastic lymphoma kinase (*ALK*) gene creates potent oncogenic drivers in patients with non-small cell lung cancer (NSCLC) occurring in approximately 3-7% of all cases. The most common fusion partner is *EML4* (echinoderm microtubule associated protein like 4) (1). In addition, at least 20 other types of fusion genes have been discovered and reported, such as *TGF-ALK*, *KIF5B-ALK*, and *STRN-ALK*. ALK+ NSCLC has been associated with the absence of smoking, younger age, and adenocarcinoma histology (2). Tyrosine kinase inhibitors (TKIs) targeting ALK have made significant breakthroughs in recent years such as extending patients’ survival periods with ALK-advanced NSCLC. To date, ALK TKIs have received approval from the US Food and Drug Administration (FDA) and European Medicines Agency (EMA) to treat advanced “ALK-positive” NSCLC. These ALK TKIs include crizotinib (first-generation), ceritinib, alectinib, brigatinib (second-generation), lorlatinib (third-generation). Clinical trials demonstrated remarkable responses within this patient population (**Table 1**). However, the clinical benefits of ALK inhibitors (ALKi) are almost universally limited by the emergence of multi-drug resistance. In this review, we analyze and summarize the mechanisms of resistance, as well as treatment strategies after resistance, in order to provide better therapeutic strategies for clinicians.

**Table 1:** Clinical trials with anaplastic lymphoma kinase inhibitors.

Drug	Clinical trials	Line of therapy	Control arm	ORR	IC-ORR	PFS(m)	OS(m)
Crizotinib	PROFILE 1005 (3)	≥2	–	54%	–	8.1	–
	PROFILE 1007 (4)	Second line	chemotherapy	–	–	7.7 *vs* 3.0	–
	PROFILE 1014 (5)	First line	Platinum doublet	74% *vs* 45%	NA	10.9 *vs* 7.0	59.8 *vs* 19.2
	PROFILE 1029 (6)	First line	Platinum doublet	87.5% *vs* 45.6%	NA	11.1 *vs* 6.8	–
Alectinib	Phase I/II (7)	≥2 crizotinib-resistant	–	22%	52%	–	–
	AF-001JP (8)	≥1 ALK-TKI-naive	–	93%	–	–	–
	Phase II (9)	≥2 crizotinib-resistant	–	50%	57%	8.9	–
	Phase II (10)	≥2 crizotinib-resistant	–	48%	52%	8.1	–
	ALUR (11)	Second line	Standard chemo	50.6% *vs* 2.5%	66.7% *vs* 0%	10.9 *vs* 1.4	–
	ALEX (12)	First line	Crizotinib	82.9% *vs* 75.5%	81% *vs* 50%	34.8 *vs* 10.4	NR
	J-ALEX (13)	First line	Crizotinib	92% *vs* 79%	NA	34.1 *vs* 10.2	68.6 *vs* 68
Ceritinib	ASCEND-4 (14)	First line	Platinum doublet	73% *vs* 2-7%	72.7% *vs* 27.3%	16.6 *vs* 8.1	NR
	ASCEND-5 (15)	Second line	Standard chemo	39% *vs* 6.9%	35% *vs* 5%	5.4 *vs* 1.6	18.1 *vs* 20.1
	ASCEND-8 (16)	a. First line b. Prior chemotherapy and/or crizotinib	450mg or 600mg with food *vs* 750 mg fasted	78.1% *vs* 75.7%	–	–	–
Brigatinib	ALTA (17)	Second line	90 mg once daily *vs* 180 mg once daily with a 7-day lead-in at 90 mg	46% *vs* 56%	50% *vs* 67%	19.6 *vs* 24.3	29.5 *vs* 34.1
	ATLA-1L (18)	First line	Crizotinib	74% *vs* 62%	78% *vs* 26%	24.0 *vs* 11.0	NR
Ensartinib	phase 1/2 trial (19)	first-line or subsequent therapy	225 mg once daily	69%	64%	9.0	–
	eXalt (20)	First line	Crizotinib	75% *vs* 67%	54% *vs* 19%	25.8 *vs* 12.7	NR
Lorlatinib	phase 2 study (21)	first-line or subsequent therapy	treatment naive (EXP1)	90%	75%	–	–
			Previous crizotinib only(EXP2)	69%	68%	–	–
			Previous crizotinib with previous chemotherapy (EXP3A)			–	–
			previous non-crizotinib ALKi, with or without chemotherapy (EXP3B)	33%	42%	–	–
			two previous ALKi with or without chemotherapy (EXP4)	39%	48%	–	–
			three previous ALKi with or without chemotherapy (EXP5)			–	–
	CROWN (22)	First line	Crizotinib	76% *vs* 58%	82% *vs* 23%	12-months:78% *vs* 39%	NR

## 2 Mechanisms of Resistance to ALK TKIs

Resistance is divided into primary and acquired resistance. Primary resistance is defined as the *de novo* lack of treatment response and can be seen after treatment with a TKI (23). While the mechanism of resistance to ALKi is less well-understood, it can be divided into two categories, on target or *ALK* dependent alterations and off target or *ALK* independent alterations.

### 2.1 *ALK* Dependent Resistance

#### 2.1.1 Secondary Mutations in the ALK Tyrosine Kinase Domain

**Figure 1:**
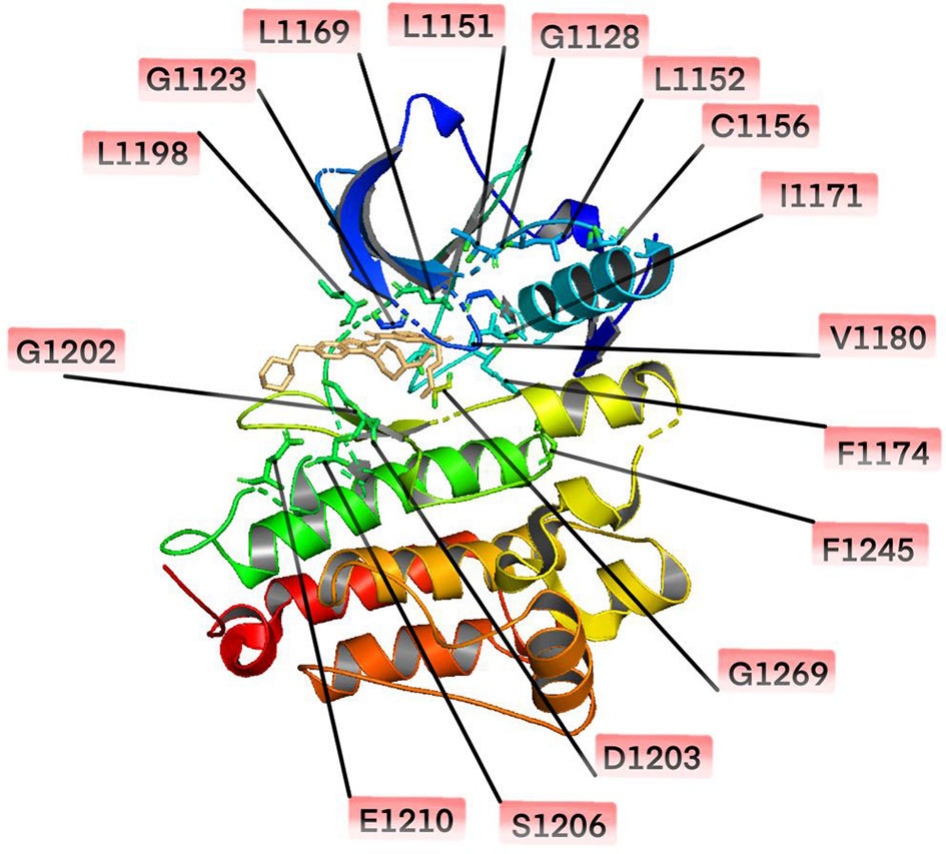
Mutations in the anaplastic lymphoma kinase (ALK) kinase domain.

**Figure 2:**
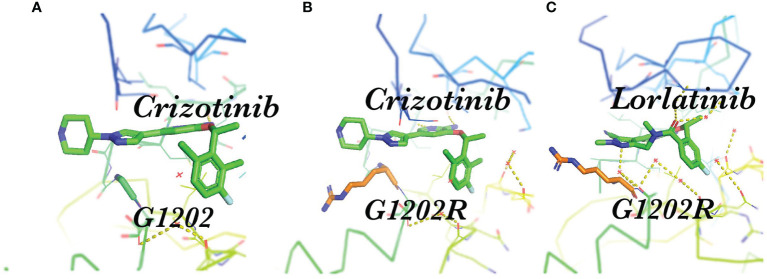
Spatial position of G1202R and ALK-TKI. **(A)** Structure of the stick representation of crizotinib (green) bound to ALK G1202; **(B)** Crizotinib bound to ALK the G1202R mutation, showing steric hindrance; **(C)** Structure of the stick representation of lorlatinib (green) bound to the ALK G1202R mutation.

Resistance mutations in the ALKi account for 30-40% of all known resistance mechanisms (24). These resistance mutations lead to structural changes in the kinase domain that interfere with the binding of the drug. A much broader spectrum of on-target mutations has been identified in ALK-positive NSCLC treated with ALK TKIs. Resistant mutations to crizotinib include L1196M、G1269A、C1156Y、G1202R、I1171T/N/S、S1206C/Y、E1210K、L1152P/R、V11180L、I1151T、G1128A、and F1174V (25–28) (**Figure 1**). The most common *ALK* mutations are mutations L1196M and G1269A, where the deep binding pocket of ATP. G1202R was found in 2% of the samples following crizotinib resistance and was the primary mechanism of second-generation ALKi resistance (**Figures 2A, B**).

After the first-generation ALK inhibitors exhibit resistance within NSCLC, many studies have shown that the sequential second-generation drugs alectinib, ceritinib, brigatinib, and ensatinib can achieve better curative effects and are superior to chemotherapy (11, 15, 29, 30). Of note, the second-generation TKIs alectinib and brigatinib are currently the preferred first-line therapies in Europe (31), while the third-generation compound lorlatinib is also approved as initial therapy by the FDA and an additional preferred first-line drug according to the current NCCN guidelines (32) (**Table 1**). The progression free survival (PFS) of alectinib was significantly better than that of crizotinib, response rate (RR) and PFS of 92% and 34.1 months, respectively. The G1202R mutation is the most common secondary resistant ALK mutant in patients post-treatment with second-generation ALK inhibitors, occurring in 21%, 29% and 43% of patients treated with ceritinib, alectinib, and brigatinib (33). It is speculated that although the second-generation ALKi have increased activity, one of the costs was the larger molecular volume of their compounds, which is heavily dependent on the direct binding to the solvent front region such as G1202 in order to increase its activity; thus, “inducing” resistance mutations within this region. Resistant mutations to alectinib include G1202R and I1171N. Tumor mutation burden and heterogeneous tumor evolution might be responsible for the rapid acquisition of alectinib resistance (34). Resistant mutations to ceritinib include G1202R、F1174V、T1151K、and T1151R (27, 35, 36), and to brigatinib include D1203N, and E1210K (37, 38).

Lorlatinib is a reversible third-generation ALK and ROS1 inhibitor that can overcome multiple *ALK* resistance mutations and penetrate the blood-brain barrier. Lorlatinib has strong activity for common mutations such as L1196M and G1269A. The G1202R mutation is particularly important as it is the primary resistance mechanism to ceritinib, alectinib, and brigatinib, whereas only lorlatinib can inhibit the *ALK* G1202R mutation (**Figure 2C**) (39). The whole exome sequencing of compound *ALK* mutations occurring in several lorlatinib-resistant patients confirms the stepwise accumulation of *ALK* mutations during sequential treatment. Several of these *ALK* kinase compound mutations that have been described include the L1196M/D1203N, F1174L/G1202R, and C1156Y/G1269A mutations (40). Absolute IC50 values of crizotinib, ceritinib, alectinib, brigatinib, and lorlatinib on cellular ALK phosphorylation in Ba/F3 cells are depicted (33). In Ba/F3 cells, *ALK* F1174C and ALK I1171T appear sensitive to ceritinib and alectinib, respectively; however, these mutations may not be susceptible to these agents *in vivo* based upon prior clinical reports. Therefore, we further combined clinical data at the cellular level in **Table 2**, which can help with medication selection after resistance. The objective response rate (ORR) was 69% in patients who had received crizotinib or crizotinib plus chemotherapy (21),which means that regardless of the previous use of several first or second-generation ALKi or chemotherapy, the efficacy of lorlatinib as a follow-up treatment is superior. Furthermore, the ORR of lorlatinib and crizotinib as a first-line therapeutic for advanced ALK+ NSCLC is 76% and 58%, revealing that lorlatinib has an advantage in regard to efficacy (22).

**Table 2:** Resistant and sensitive mutations of 6 ALK inhibitors (S, sensitive; R, Resistance).

Variant	Crizotinib	Ceritinib	Ensartinib	Alectinib	Brigatinib	Lorlatinib
G1123S	S	R (41)	S	S (41)	S	S
I1151Tins	R (24)	R	S	S	S	S
L1152P	R	R	S	S	S	S
L1152R	R (42)	R (43)	S	S	S	S
C1156T	R	R	S	S	S	S
C1156Y	R (44)	R (45)	S	S	S	S (45),R (37)
I1171N	R (33)	S (46)	S	R (27)	S (47)	S,R (48)
I1171T	R (49)	S (46)	S	S,R (33)	S	S
I1171S	R (33)	–	–	R (33)	S (50)	–
F1174C	R (51)	S,R (33)	R	S (52)	S	S
F1174L	R (49)	S,R (33)	S	S	S	S
F1174V	R (52)	S,R (27)	R	S (52)	S	S
V1180L	R	S (53)	S	R (33)	S (47)	S
L1196M	R (44)	S (40),R (33)	S	S,R (33)	S (47)	S
L1198F	S (45)	R	S	R	R	R (45)
L1198P	R (54)	–	–	–	–	–
G1202R	R (24)	R (27)	R (55)	R (27)	R (56),S (47)	S (56)
D1203N	R (54)	R (40)	R	R	R (37)	R (40)
S1206C	R	S	S	S	R	S
S1206Y	R (24)	S (57)	S	S	S	S
E1210K	R (49)	R	R (55)	R	R (38)	S
E1407K	–	–	–	–	R (37)	–
F1245C	R (58)	R,S (58)	R	R	R	S
F1245V	R (27)	–	–	S (27)	–	–
G1269A	R (59)	S	S,R (55)	S	S	S
G1269S	R (54)	S	R	S	S	S
G1123D	–	–	–	–	–	R (37)

When patients receive sequential ALKi treatment, the cancer cells accumulate new mutations in addition to the previously acquired mutations, making treatment more complex (33, 37, 40, 60). Takahashi, Ken reported a patient who underwent sequential treatment with crizotinib, alectinib and lorlatinib; thus, developing the double mutations I1171S and G1269A. Ceritinib and brigatinib have the potential to become the therapeutic agents to treat this double mutation (61). Geeta G. Sharma’s team reported a case of ALK-positive NSCLC with the dual mutation *ALK* L1196M/G1202R after brigatinib treatment. Lorlatinib was effective against the G1202R mutation. Interestingly, this patient’s L1196M/G1202R dual mutation also increased primary resistance to lorlatinib, further limiting treatment options (62). ALK D1203N was significantly more common at relapse with lorlatinib than second-generation ALKi’s (63). In one case of ALK-positive NSCLC, after the failure of continuous treatment with crizotinib and alectinib, the mutation of the *ALK* fusion gene L1196M was detected, and no other acquired drug resistance mechanism was found. The patient developed resistance to alectinib, but remained sensitive to ceritinib (64).

**Figure 3:**
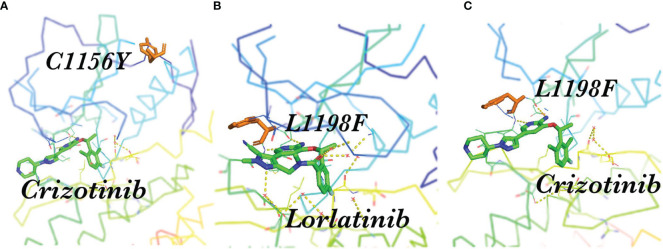
Spatial position of C1156Y, L1198F, and ALK-TKI. **(A)** Crizotinib and the resistance mutation C1156Y in the ALK kinase region; **(B)** L1198F developed resistance to lorlatinib through the spatial interference of drug binding; **(C)** L1198F enhanced ALK kinase domain binding to crizotinib.

However, not all complex mutations increase the difficulty of treatment (**Table 3**). Interestingly, some compound mutations that lead to lorlatinib resistance led to re-sensitization of the first or second generation ALKi (65). A patient receiving sequential treatment for ALK-positive NSCLC was resistant to crizotinib due to the mutation C1156Y in the ALK kinase region (**Figure 3A**). Sequencing revealed the mutation *ALK* L1198F in addition to C1156Y (**Figure 3B**). The L1198F mutation developed resistance to lorlatinib through spatial interference with drug binding. However, the L1198F mutation enhanced its binding to crizotinib (**Figure 3C**), making it sensitive to the C1156Y mutation. The patient was treated again with crizotinib, resulting in the successful treatment of cancer-related symptoms and liver failure (45). Other researchers have also demonstrated that the L1198F mutation leads to conformational changes in the inhibitor site as well as changes in the binding affinity of ALK to crizotinib and lorlatinib (66).

**Table 3:** Compound mutations and Treatment recommendations.

Team	Previous treatment	Compound mutation	Note
	Crizotinib,alectinib,lorlatinib	I1171S+G1269A	Recommended drugs: ceritinib, brigatinib
Shaw AT et al (45)	Crizotinib, lorlatinib	C1156Y+L1198F	re-sensitization: Crizotinib
Okada K et al (65)	Alectinib, lorlatinib	I1171N+L1256F	re-sensitization: Alectinib
Okada K et al (65)		I1171N+L1198F	Compound mutations are more sensitive to crizotinib than I1171N single mutants

For patients with drug resistance after ALK-TKI treatment, a re-biopsy is recommended to provide optimal treatment. Haratake N et al. retrospectively analyzed ALK-TKI treatment patterns and clinical outcomes. Of the 71 patients treated with ALK-TKI for NSCLC, 20 were re-biopsied, and 8 had secondary drug-resistant mutations. The ORR of patients with ALK point mutations receiving ALK-TKI was 88.9%, while patients without the ALK point mutations receiving ALK-TKI or chemotherapy were only 20.0%. However, PFS in patients with secondary drug-resistant mutations are relatively short, and their mechanism needs to be further studied (67).

#### 2.1.2 Amplification of *ALK*

*ALK* amplification occurs at a low frequency, but it is responsible for acquired resistance to crizotinib. Katayama R reports a high level of *ALK* amplification in 15 NSCLC patients with crizotinib resistance (24).

### 2.2 *ALK*-Independent Resistance

#### 2.1.1 Activation of Bypass Signaling Pathways

**Figure 4:**
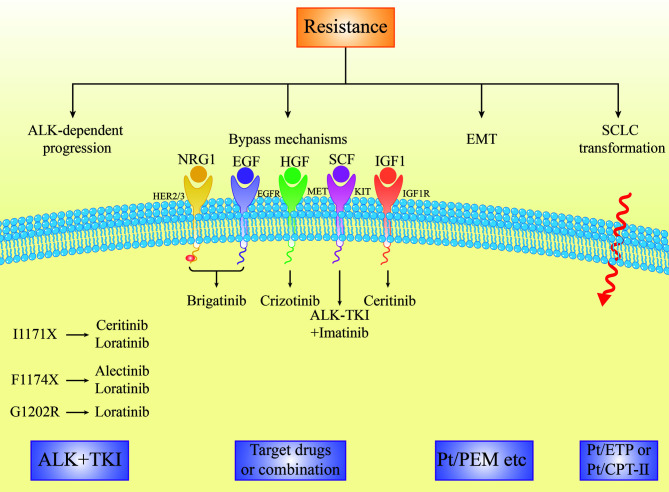
The resistance mechanisms of ALK TKIs in advanced NSCLC and next treatment strategy.

Activation of the bypass signaling pathways is the resistance mechanism of ALK-TKIs, including *EGFR* signaling (42, 68), amplification of *KIT* (24), *IGF-1R-IRS-1* pathway (69), *MAPK* (70), *MET* amplification (71–73), *BRAF V600E* mutation (73), and the activation of the transcriptional co-regulator YAP (74) (**Figure 4**). In addition, Recondo G et al. found a new bypass mechanism caused by drug resistance due to NF2 functional deletion mutations, increasing mTOR inhibitor treatment sensitivity (40). Bypass activation is more common in patients with sequential TKI than in patients with crizotinib alone (49).

The activation of the EGFR pathway is one of the mechanisms of ALK-TKI resistance, such as crizotinib and alectinib. Ceritinib and afatinib combinatorial treatment partially restored the sensitivity to ceritinib (68). Afatinib may be a promising treatment for overcoming ceritinib resistance in ALK or ROS1-positive NSCLC cells by inhibiting the neuroregulatory protein (NRG1) signaling pathway (75).

Increased expression of hepatocyte growth factor (HGF) and its physiological receptor tyrosine kinase MET is associated with acquired resistance to various TKIs. *MET* amplification was detected in 12% and 22% of biopsies of patients using second-generation inhibitors or lorlatinib, respectively. Patients treated with second-generation ALKi during first-line therapy were more likely to have *MET* amplification than those treated with second-generation ALK inhibitors after crizotinib treatment (76). Gab1 is a key effector in the HGF/MET signaling pathway that mediates alectinib resistance. The antidiabetic drug metformin combined with alectinib overcomes HGF/Met-induced alectinib resistance by blocking the complex formation between MET and Gab1, thus inhibiting Gab1 phosphorylation and activating the downstream signaling pathway. These results suggest that metformin combined with alectinib may help overcome alectinib resistance caused by the HGF/MET signaling pathway activation, improving the efficacy of alectinib (77).

Cerivastatin, a rate-limiting enzyme inhibitor of the mevalonate pathway, showed anticancer activity against ALK-TKI *in vitro* and *in vivo*, accompanied by inactivation of the transcription-assisted regulator YAP. Cerivastatin can significantly induce YAP-targeted oncogenes (EGFR, AXL, CYR61 and TGFbetaR2) in drug-resistant cells, providing a theoretical basis using YAP as a potential therapeutic option in patients with acquired drug-resistant ALK-TKI (74).

#### 2.2.2 Drug Efflux Pump

P-glycoproteins (P-gp) are highly conserved ATP-dependent effluents encoded by the multidrug resistance 1(*MDR1*) gene, also known as the ATP binding box subfamily B member 1(ABCB1) (78). The central nervous system (CNS) is the primary site of failure in most patients with crizotinib resistance. P-gp efflux and limited diffusion of crizotinib results in limited blood-brain barrier penetration (79). In contrast, alectinib is not a P-gp substrate and can achieve higher CNS levels (80, 81).

#### 2.2.3 Lineage Changes

Morphological changes are also one of the mechanisms of ALK-TKI resistance in NSCLC. Many cases have reported drug resistance due to the conversion to small cell lung cancer (SCLC) or squamous cell carcinoma (SCC) after targeted therapy against ALK-positive adenocarcinoma (82–86). Deletion of p53 and retinoblastoma (*RB*) genes is important for SCLC transformation, although the transformation mechanism is not fully understood (87). Mutations in the *TP53* and *PTEN* genes were also found in a patient with SCLC transformation (88). In addition, a patient who underwent alactinib treatment developed transformed SCLC. The levels of gastrin-releasing peptide precursor and neuron-specific enolase in the patients were increased, indicating SCLC transformation during the drug resistance of ALK-tyrosine kinase inhibitors (83).

In addition to the conversion of adenocarcinoma to SCC or SCLC, Koyama K. et al. reported a rare case of ALK-positive adenocarcinoma that converted to NSCLC with neuroendocrine differentiation. Histopathological examination of the tumor following alectinib resistance revealed a poorly differentiated carcinoma with insulinoma associated protein 1 (INSM1) expression. The expressions of CD133, Bcl-2, and SOX2 were positive when compared with the initial tumor. SOX2 expression was significantly increased compared to that before treatment. Immunohistochemical results of these markers associated with tumor stem-like cells and neuroendocrine differentiation suggest that tumor stem cells play a role in the histological transformation and acquired resistance mechanisms of ALK-reposition-positive tumors (89). HER2 plays an important role in regulating cancer stem cell phenotypes of ALK translocation lung cancer, which is primarily mediated by HER2/HER3 heterodimers (90).

Epithelial-to-mesenchymal transition (EMT) is a morphological change in which epithelial cells lose their polarity and intercellular connections becoming more mobile and invasive. Through EMT, tumor cells acquire mesenchymal morphology and the ability to migrate and invade. There are four pathways associated with EMT: proteoglycan in cancer, HIF-1 signaling, FoxO signaling, and extracellular matrix receptor interactions, related to the drug resistance mechanisms of crizotinib (91) (**Figure 4**). *ALK* mutants L1196M and EMT were simultaneously detected in a patient with crizotinib resistance. *ALK* L1196M primarily existed within the epithelial tumor cells, suggesting that EMT and *ALK* mutations co-exist as independent mechanisms of drug resistance. EMT was associated with decreased expression of miR-200c and increased expression of ZEB1, leading to cross-resistance of the new generation of ALKi. The histone deacetylase (HDAC) inhibitor overcomes this resistance by reversing EMT *in vitro* and *in vivo*, suggesting that adding a new ALKi after pretreatment with an HDAC inhibitor may help overcome the co-occurrence of ALK resistance mutations and EMT (92).

Kang, J. et al. performed next generation gene sequencing (NGS) on 42 crizotinib-resistant NSCLC patients. Two patients were found to have acquired mutations in the DNA mismatch repair gene *POLE*, leading to a significant increase in the tumor mutation burden, possibly leading to a poor response to crizotinib (93). Lai, Y. et al. investigated the resistance of microRNAs (miRNAs) to the ALK TKIs NSCLC cell lines. It was found that miR-100-5p makes *EML4-ALK* NSCLC cells resistant to crizotinib and lorlatinib, and maybe a therapeutic target for drug resistance (94).

The expression of the ATP-binding domain C-member 11 (ABCC11) in alectinib-resistant cell lines was significantly higher than that in alectinib-sensitive cell lines. This indicated that ABCC11 expression may be involved in the acquired drug resistance of alectinib (95). In addition, the neuroregulatory peptide U (NMU) may make NSCLC resistant to alectinib (96).

### 2.3 Primary ALK TKI Resistance

Progression of ALK-TKI within 3 months is considered primary resistance. In theory, any of these mechanisms of acquired resistance that existed before the use of TKI could also lead to primary resistance (23).

BIM is a Bcl-2 (B lymphocytoma-2) -like protein 11 that activates programmed cell death in cells. The study found that patients with BIM with missing polymorphisms had shortened PFS and reduced objective response rate, which was an independent predictor of patients treated with crizotinib and was related to primary drug resistance (97). In addition, the low minimum allele frequency (MAF) of the *EML4-ALK* rearrangement may also be a mechanism of primary resistance to crizotinib (98).

Rihawi K reported a patient with primary resistance to crizotinib. *MYC* amplification was a potentially new mechanism of primary ALK-TKI, resistance and proposed as a potential MYC-oriented inhibition strategy to overcome primary resistance of advanced *ALK*-rearrangement NSCLC (99). Similarly, the results of Pilling AB et al. showed a dual oncogene mechanism, in which ALK positively regulates the MYC signaling axis, providing an additional oncogene target (100).

## 3 Discussion

The treatment of ALK-rearranged NSCLC with ALK TKIs has significantly changed these patients’ outcome and quality of life. However, all patients will inevitably progress in time. Clinicians use imaging to determine whether a patient is resistant so that, if possible, they can switch to the next generation of ALK-TKI quickly. However, caution should be exercised in judging disease progression, as radiological progression may either be non-tumor cell proliferation and/or accumulation (101). In patients who have received radiation therapy, sequential ALK-TKI should be recognized as radionecrosis of the central nervous system, since treatment with the next generation of ALK-TKI may increase its severity (102).

The brain is the primary site of failure with ALK inhibitors in ALK-positive patients and is considered a sanctuary site owing to the blood–brain barrier (BBB) (103, 104). *ALK*-rearranged NSCLC patients exhibiting a history of prior ALKi treatment are reported to harbor a high incidence of CNS metastases, i.e., from approximately 45 to 70%, suggesting that brain metastasis is the most common form of failure with ALKi therapy. A limitation of crizotinib is that relapse in the brain after treatment was commonly reported (104). Next-generation ALK inhibitors were designed to pass the BBB. The time to CNS progression was significantly longer with alectinib than with crizotinib (cause-specific hazard ratio, 0.16, 95% CI, 0.10 to 0.28; rate of events of CNS progression, 12% with alectinib and 45% with crizotinib), which is attributed to the expression of P-gp’s on the luminal side of the BBB endothelium (9, 80, 105, 106).

Acquired resistance has become an important issue. Previous investigations additionally presented the *in vitro* IC50 values for all available ALK TKIs regarding the different mutations. The findings illustrate that lorlatinib has the broadest activity against the G1202R mutation (33, 107). However, whether ALKi is sensitive or resistant is complex within the real world. For example, G1202R has been detected in biopsy specimens from patients with *ALK*-rearranged NSCLC who relapsed on brigatinib, suggesting that its potency may be compromised with this mutation; however, some cases were effective with brigatinib treatment. This may result from the steric hindrance between the side chain of G1202R and the extended solubilization group of brigatinib (**Figure 2**)

The fusion variant background should also be taken into consideration when interpreting *ALK* resistance mutations. Among>15 *EML4-ALK* variants have been identified to date, the five most common variants are variant 1 (v1; E13, A20), variant 2 (v2; E20, A20), variant 3 (v3; E6, A20), variant 4 (v4; E15, A20), and variant 5 (v5; E2, A20). The two *EML4-ALK* variants that together account for up to 70-80% of all *EML4-ALK* variants are v1 and *EML4-ALK* v3a/b (108). The ALTA-1L analysis by variants was the first validation of the significance of EML4-ALK variants in the context of a prospective randomized phase 3 study (109). **Table 4** shows the differences in PFS between variants 1 and 3. That suggests that the *ALK* fusion variant may affect clinical outcomes. The reason for this difference may be relatively stable in short EML4-ALK variants, which leads to accumulation and stronger carcinogenic signaling, and their better interactions with cell skeletons, which increases the migration capabilities of V3-positive cancer cells (117, 118). However, the molecular basis for this association is unknown. Besides, TP53 mutations and V3 are independently associated with enhanced metastatic spread, shorter TKI responses and inferior overall survival in ALK positive lung adenocarcinoma (115). Furthermore, *ALK* resistance mutations were significantly more common in variant 3 than in variant 1 (57% v 30%; P = .023). In particular, the *ALK* G1202R mutation was more common in variant 3 than in variant 1 (32% v 0%; P <.001). Among the patients treated with the third-generation ALK TKI lorlatinib, variant 3 was associated with a significantly longer progression-free survival than variant 1 (hazard ratio, 0.31; 95% CI, 0.12 to 0.79; P = .011) (112, 119). These results suggest that among the *EML4-ALK* v3 patients, we should consider introducing more aggressive therapies earlier on in the course of the disease (**Table 4**).

**Table 4:** List of retrospective analyses comparing clinical efficacy of EML4-ALK variants and ORR and PFS in prospective phase 3 trial of first-line ALK TKIs.

		V1		V3	P value	References
ORR		72.7%		55.6%	0.214	Lei et al., Clin Lung Cancer 2016 (110)
PFS		11m		10.9m	0.795	
PFS (crizotinib)		11.5m		8.7m	0.18	Noh et al., J Path 2017 (111)
PFS (1st line crizotinib)		8.9m		6.9m	0.163	Lin et al., JCO 2018 (112)
PFS (2nd-generation ALK TKIs post-crizotinib)		11.8m		7.9m	0.141
PFS (lorlatinib post crizotinib and 2nd-generation ALK TKIs)		3.3m		11m	0.011
PFS		7.9m		11.9m	0.285	Kron et al., Ann Oncol 2018 (113)
PFS (after 1st-line ALK TKIs)		V1/2	39.3m	7.3m	0.01	Christopoulo et al., Int J Cancer 2018 (114)
PFS (after 1st-line chemotherapy)			15.2m	5.4m	0.008
OS			59.6m	39.8m	0.017
PFS (after 1st-line ALK TKIs)			16m	7m	0.031	Christopoulo et al., Int J Cancer 2019 (115)
PFS (after all lines of ALK TKI)			10m	7m	0.003
OS			59m	35m	0,026
PFS (crizotinib-treated)		12.2m		12.3m	0.2697	Li et al., Frontier Oncology 2020 (116)
PFS (baseline brain mets, crizotinib-treated)		10.7m		12.39m	0.6274
crizotinib	ORR	66.7%		45.8%	0.2959	ALEX
	PFS	7.4m		9.1m	0.8504	
alectinib	ORR	90.5%		83.3%	0.3538	ALEX
	PFS	34.8m		17.7m	0.4226	
brigatinib	ORR	84%		84%	–	ATLA-1L (109)
	PFS	NA		16m	–	
ALK resistance mutations		30%		57%	0.023	Lin, J. J., J Clin Oncol 2018 (112)
G1202R		0		32%	0.001
G1202R		lorlatinib		lorlatinib	–	Horn, L., et al. J Thorac Oncol 2019 (107)
G1202Rdel		brigatinib		brigatinib	–
G1269A		brigatinib		brigatinib	–

Interestingly, more patients are using second-generation TKIs. However, in this final J-ALEX OS analysis, prolongation of OS in the alectinib arm was not observed compared to the crizotinib arm. This indicates that longer PFS does not translate into longer OS, which gives clinicians something to think about when using ALKi’s. Therefore, there is much debate about whether PFS can be converted to OS, due to the following reasons. First, after the PFS benefit, disease progression may be faster than in the control group (120). Second, in a study that included 14 studies (N = 12567) in patients with advanced NSCLC submitted to the FDA between 2003 and 2013, a logarithmic scale scatter plot of the therapeutic effects showed no association was observed between PFS and OS in all studies (14, including the targeted studies) (R2 = 0.08; 95% CI 0-0.31) (121).

To expand PFS and OS, it is crucial to think about evidence-based treatment sequencing. Every ALK TKI has its own advantages and disadvantages (122). Therefore, a second biopsy is recommended for gene sequencing when the patient is resistant (123). However, repeated tumor biopsies to identify secondary resistance mutations are invasive and in certain cases not feasible. New tools are needed to evaluate tumor heterogeneity better and monitor tumor mutational profiles over time and throughout disease evolution (124, 125). Circulating tumor DNA (ctDNA) can be used as a strategy to identify therapeutic response and drug resistance (107). In contrast to ctDNA, circulating tumor cells (CTC) are either apoptotic or alive, but viable CTCs contain tumorigenic cell clones with high relevance for metastatic progression (126). Besides, copy number variation (CNV) profiling and targeted panel sequencing from cell-free DNA (cfDNA) were also performed to monitor ALK+ NSCLC (127).

Approximately30% of ALK-positive NSCLC patients resistant to crizotinib are related to secondary *ALK* mutations or amplification. Therefore, the next generation of ALK-TKIs becomes sensitive to some mutations. However, nearly 40% of patients with second-generation TKI resistance are no longer dependent on *ALK*, so treatment opportunities for these patients are limited. Third-generation ALK-TKI or pemetrexed-based chemotherapy may be beneficial making loratinib more effective in patients with ALK kinase domain point mutation than those without *ALK* re-mutation (128). An effective long-term strategy may be to pre-treat with third-generation ALK-TKI in order to prevent the emergence of resistance (129). The use of immunotherapies for ALK-TKI is still lacking (130, 131). Although patients with advanced NSCLC showed a good response to immune checkpoint inhibitors, this was associated with high PD-L1 expression levels, a high mutant load, and a history of smoking (132). However, ALK-positive patients tend not to smoke, have a low tumor mutation load (133), and have a poor response to PD-1 inhibition (134). Positive PD-L1 expression was associated with unfavorable clinical outcomes in patients with ALK-positive lung adenocarcinoma receiving crizotinib (135). ALK-positive tumors progressing with ceritinib therapy are not immunogenic enough to respond to immune checkpoint inhibitors (136). However, a successful pembrolizumab treatment case of lung adenocarcinoma after becoming resistant to ALK-TKI treatment due to G1202R mutation was reported (137). Therefore, the potential benefits of adding immunotherapy to ALK TKI therapy remains unclear.

Activation of bypass signals has emerged as another potential strategy for combating ALK-TKI resistance. Leptomeningeal Carcinomatosis (LMC) often occurs in ALK-positive NSCLC. EGFR bypass activation is known to be the drug resistance mechanism against ALK-TKI therapy. EGFR-TKI *in vitro* resensitizes cells to alectinib and successfully controls the progression of LMC, indicating the therapeutic potential of new therapies targeting both ALK and EGFR for ALK-TKI resistant LMC (138). In addition, apatinib can restore sensitivity to alectinib by inhibiting the downstream ALK and anti-angiogenic signaling pathway. Furthermore, reversing ALK-TKI and inhibiting angiogenesis in combination with alectinib and apatinib, thus inhibits ALK and VEGF R2 controlling the progression of the *EML4-ALK* fusion gene lung cancers (139). Furthermore, PFS was more severe in patients with TP53 co-mutations than in patients with wild-type *TP53*, meaning the combination of proteasome inhibitors with alectinib is a promising therapy for NSCLC with *ALK* rearrangement/*TP53* mutations (49, 140).

## 4 Conclusion

Despite the significant efficacy of ALKi in ALK-positive NSCLC patients, drug resistance is inevitable in some patients. Although the mechanism of drug resistance can be divided into *ALK*-dependent and non-dependent, the specific mechanisms have not been clarified, so there is urgency in developing strategies to overcome or prevent drug resistance. With a growing understanding of the mechanisms of drug resistance, a new generation of ALKi is expected to be more effective in overcoming and suppressing drug resistance. After drug resistance, it is recommended to biopsy again to identify the mutation site. Moreover, variants should also be of concern. In addition, combination therapy is also an option. However, there may be potential problems of increased toxicity or emergence of new toxicities, so these combinatorial treatment regimens still need to be explored. Furthermore, there is much debate about whether PFS can be converted to OS. In targeted therapy, it depends on the PFS1, 2, and 3. In patients with *ALK* fusion, the first generation may be followed by second generation therapy, or the second generation is followed by another second generation therapeutic. In short, these new approaches are promising at more effectively overcoming and suppressing drug resistance, translating into more profound and more prolonged responses in patients with *ALK*-driven cancers.

## Author Contributions

YP participated in the analysis, data interpretation, and wrote the manuscript. CD and ZQ polish the language and search the literature. CC analyzed the data and drew diagrams. FW designed the article ideas analyzed the data. All authors contributed to the article and approved the submitted version.

## Conflict of Interest

The authors declare that the research was conducted in the absence of any commercial or financial relationships that could be construed as a potential conflict of interest.

## Publisher’s Note

All claims expressed in this article are solely those of the authors and do not necessarily represent those of their affiliated organizations, or those of the publisher, the editors and the reviewers. Any product that may be evaluated in this article, or claim that may be made by its manufacturer, is not guaranteed or endorsed by the publisher.
